# Landscape of Immune Microenvironment Under Immune Cell Infiltration Pattern in Breast Cancer

**DOI:** 10.3389/fimmu.2021.711433

**Published:** 2021-08-27

**Authors:** Qianhui Xu, Shaohuai Chen, Yuanbo Hu, Wen Huang

**Affiliations:** ^1^Department of Nephrology, The Second Affiliated Hospital and Yuying Children’s Hospital of Wenzhou Medical University, Wenzhou, China; ^2^Department of Surgery, The Second Affiliated Hospital, School of Medicine, Zhejiang University, Hangzhou, China

**Keywords:** breast cancer, tumor immune microenvironment, immune cell infiltration, immunotherapy, prognosis

## Abstract

**Background:**

Increasing evdence supports the suggestion that the immune cell infiltration (ICI) patterns play a pivotal role in tumor progression in breast cancer (BRCA). Nonetheless, there has been no comprehensive analysis of the ICI patterns effects on the clinical outcomes and immunotherapy.

**Methods:**

Multiomic data for BRCA samples were downloaded from TCGA. ESTIMATE algorithm, ssGSEA method, and CIBERSORT analysis were used to uncover the landscape of the tumor immune microenvironment (TIME). BRCA subtypes based on the ICI pattern were identified by consensus clustering and principal-component analysis was performed to obtain the ICI scores to quantify the ICI patterns in individual tumors. Their prognostic value was validated by the Kaplan-Meier survival curves. Gene set enrichment analysis (GSEA) was applied for functional annotation. Immunophenoscore (IPS) was employed to explore the immunotherapeutic role of the ICI scores. Finally, the mutation data was analyzed by using the “maftools” R package.

**Results:**

Three different immune infiltration patterns with a distinct prognosis and biological signature were recognized among 1,198 BRCA samples. The characteristics of TIME under these three patterns were highly consistent with three known immune profiles: immune- excluded, immune-desert, and immune-inflamed phenotypes, respectively. The identification of the ICI patterns within individual tumors based on the ICI score, developed under the ICI-related signature genes, contributed into dissecting biological processes, clinical outcome, immune cells infiltration, immunotherapeutic effect, and genetic variation. High ICI score subtype, characterized with a suppression of immunity, suggested an immune-exhausted phenotype. Abundant effective immune cells were discovered in the low ICI score patients, which corresponded to an immune-activated phenotype and might present an immunotherapeutic advantage. Immunophenoscore was implemented as a surrogate of immunotherapeutic outcome, low-ICI scores samples obtained a significantly higher immunophenoscore. Enrichment of the JAK/STAT and VEGF signal pathways were activated in the ICI low-score subgroup. Finally, the synergistic effect between the ICI score and the tumor mutation burden (TMB) was confirmed.

**Conclusion:**

This work comprehensively elucidated that the ICI patterns served as an indispensable player in complexity and diversity of TIME. Quantitative identification of the ICI patterns in individual tumor will contribute into mapping the landscape of TIME further optimizing precision immunotherapy.

## Introduction

Globally, breast cancer (BRCA) is one of the most frequently diagnosed malignant tumors and is the first leading cause of tumor-related death in females (1–3). Based on the latest global cancer statistics, there were almost 2.1 million newly diagnosed BRCA cases in 2018 worldwide (1). Early diagnosis difficulties and rapid tumor progression mean that a majority of BRCA patients presented with an advanced disease or metastatic lesions at diagnosis (4). Therefore, it is important to generate robust tools for prognosis prediction and therapeutic response assessment because these would further facilitate precision and individualized treatment.

Cancer immunotherapy harnesses an anti-tumor immune response to recognize and eliminate tumor cells by activating the host immune system. Although T cell-related immune responses induce anti-tumor responses by increasing immune checkpoint inhibitors, only a minority of cancer patients benefit from them (5). It was well established that BRCA was considered as tumor of immunologically quiescent, which greatly held back its therapeutic response to immunotherapy. The encouraging results of preclinical trials and recent clinical data indicated that an immunotherapeutic strategy may be the key to unlock the new era of clinical intervention in BRCA (6, 7). Besides, accumulating evidences supported that immune infiltration in the tumor immune microenvironment (TIME) functioned as decisive players for predicting the prognosis of BRCA (8). Recent researches reported that the number of T cells had a significant association with the prognosis in patients with triple-negative breast cancers (9, 10). Hence, the most reliable and promising approach for a comprehensive evaluation of tumor sensitivity to immunotherapy may be the one derived from the immune cell infiltration (ICI) profiles, clustering BRCA samples based on molecular-specific subgroups associated with the ICI patterns, facilitating personalized tailored treatment, and increasing therapeutic benefits accordingly. However, researches focused on the comprehensive contexture of ICI patterns-mediated TIME of BRCA which is lacking.

In this work, the underlying interactions of the ICI patterns with context of TIME were comprehensively analyzed by employing the genomic and transcriptomic information of 1,198 BRCA samples from the TCGA-BRCA project and GSE58812 datasets. CIBERSORT algorithm, ssGSEA algorithm, and ESTIMATE algorithm were employed to draw the landscape of TIME by analyzing the genomics information of BRCA. Three different ICI patterns subtypes were determined by using consensus clustering, and the characterization of TIME under distinct ICI patterns could be considered as different immune phenotypes: immune-excluded, immune-desert, and immune-inflamed, respectively. In addition, ICI-based scoring scheme was established to identify the ICI patterns of individual samples and estimate the immunotherapeutic response. Moreover, response to immunotherapy in different ICI scores samples were predicted to contribute promising insights into advance precision immunotherapy. Finally, both the intrinsic connection and synergistic effect of the ICI score with the tumor mutation burden (TMB) was demonstrated. In summary, our results indicated that the ICI patterns functioned as indispensable roles in the formation of diversity and complexity of TIME and contributing into tailored immunotherapeutic strategy in BRCA.

## Materials and Methods

### Breast Cancer Data Collection

The breast cancer RNA-sequencing transcriptomic data and the corresponding clinical profiles were procured from The Cancer Genome Atlas (TCGA) database and the Gene Expression Omnibus (GEO). A total of 1,198 BRCA samples were employed for subsequent analysis. The gene-expression profiles of TCGA-BRCA in the Fragments Per Kilobase per Million (FPKM) format were obtained from the TCGA portal (http://cancergenome.nih.gov), and then were transformed into TPMs (transcripts per kilobase million). The microarray dataset GSE58812 were downloaded from the GEO database (https://www.ncbi.nlm.nih.gov/geo/). To reduce the likelihood of batch effects from non-biological technical biases between different datasets, the “ComBat” algorithm was applied (11).

### Landscape of Immune Cells Infiltration in the Tumor Immune Environment

By using the CIBERSORT package (http://cibersort.stanford.edu/), the gene expression information of the TCGA and GEO cohorts was analyzed to obtain a fraction matrix of ICI, which estimate the abundances of 22 distinct leukocyte subsets (12). The Estimation of Stromal and Immune Cells in Malignant Tumors using the Expression Data (ESTIMATE) algorithm (13) takes advantage of the unique properties of the transcriptional profiles to infer the tumor cellularity as well as the tumor purity. By using the ESTIMATE approach, the ESTIMATE, immune, and stromal scores were calculated to predict the level of infiltrating immune and stromal cells and these forms the basis to infer tumor purity. Besides, single sample gene-set enrichment analysis (ssGSEA) was conducted based on the expression level of 29 immunity-associated signatures using the R package “GSEAbase”.

### Consensus Clustering for Tumor Immune Cell Infiltration

To further functionally reveal the biological significance of immune cell infiltration in BRCA, the “ConsensusClusterPlus” package (http://www.bioconductor.org/) were employed to stratify the BRCA samples into three discrete subgroups, with a hierarchical agglomerative consensus, based on the ICI matrix of each sample. The unsupervised clustering “Pam” method based on Euclidean and Ward’s linkage was used in this analysis and repeated 1,000 times to ensure the classification stability.

### DEGs Among Inter-Immune Cell Inflitration Clusters and Enrichment Analysis

Based on the previous consensus clustering algorithm, samples were clustered into the ICI subgroups to determine the genes correlated with the ICI patterns. Next, ICI-related differentially expressed genes (DEGs) were determined among these ICI patterns by using the ‘limma’ R package. The DEGs with an adjusted P-value < 0.05 and absolute fold-change > 1.5 were considered as significant and employed for further analysis. To further elucidate the biological role of DEGs, Gene ontology (GO) annotation was conducted.

### Development of the Immune Cell Infiltration Scoring Scheme (ICI Scores)

First, unsupervised clustering was conducted to classify BRCA patients as per the DEG values. Next, DEGs values that were positively and negatively correlated with the clusters signature were considered as the ICI gene signatures A and B, respectively. Furthermore, the Boruta algorithm was carried out for the dimension reduction of the ICI gene signatures A and B, and principal component 1 was fetched as the signature score by using the principal-component analysis (PCA). Finally, the ICI score A based on ICI signature gene A and the ICI score B based on ICI signature gene B were aggregated into a prognostic score, which was defined as the ICI score. We applied a method similar to the Gene expression grade index to define the ICI score of each patient: ICI score = ∑PC1A-∑PC1B.

### Role of the Immune Cell Infiltration Score in the Tumorigenesis Features

Gene set enrichment analysis (GSEA) was performed to functionally elucidate the biological roles of the ICI score in BRCA. We analyzed the gene sets of “c2.cp.kegg.v7.2.symbols.gmt [Curated]” from the Molecular Signatures Database through GSEA (14). To achieve a normalized enrichment score for each analysis, gene set permutations with 1,000 times were carried out. A nominal p < 0.05 and FDR q < 0.05 were regarded as significant results.

### Process of Epigenetic Mutation Data

The corresponding somatic alteration information of the TCGA-BRCA cohort were obtained from the TCGA dataset. TMB was defined as the number of somatic, coding, base replacement, and insert-deletion mutations per megabase of the genome examined using non-synonymous and code-shifting indels under a 5% detection limit. The “maftools” R package (15) was used to calculate the number of somatic non-synonymous point mutations within each sample.

### Correlation of the Immune Cell Infiltration Score With Immunotherapy

Refer to existing studies, the expression level of immune checkpoint blockade-related key genes might be correlated with the clinical outcome of immune checkpoint inhibitors blockade treatment (16). Herein, we employed six key genes of immune checkpoint blockade therapy: programmed death ligand 1 (PD‐L1, also known as CD274), programmed death ligand 2 (PD‐L2, also known as PDCD1LG2), programmed death 1 (PD‐1, also known as PDCD1), cytotoxic T‐lymphocyte antigen 4 (CTLA‐4), indoleamine 2,3‐dioxygenase 1 (IDO1), and T‐cell immunoglobulin domain and mucin domain‐containing molecule‐3 (TIM‐3, also known as HAVCR2) in BRCA (17–19). To elucidate the potential player of the ICI score in immunotherapy, the correlation of the ICI score with an expression level of six immune checkpoint blockade key genes was analyzed. Furthermore, we determined and compared the expression value of 15 immune checkpoint blockade-related genes (i.e., PDCD1, etc.) between patients with a low-/high-score. Immunophenoscore (IPS) refers to four main parts (effector cells, immunosuppressive cells, MHC molecules, and immunomodulators) determining the immunogenicity, and is calculated without bias using machine learning methods. The IPS (ranges 0–10) is calculated based on the gene expression in representative cell types. It has been verified that IPS could predict the response of the patients to immunotherapy (20). The IPS of BRCA patients were downloaded from The Cancer Immunome Atlas (TCIA) (https://tcia.at/home).

### Construction of the Prognostic Nomogram

To provide a scoring system to predict the prognosis quantitatively, a prognostic nomogram that consists of the ICI score and clinical variables was developed to estimate the 3‐, 5‐, and 10‐year OS possibilities. Additionally, the calibration curve which could validate the prognostic value of the as-constructed nomogram was analyzed.

### Statistical Analysis

The Wilcoxon test was employed to compare two groups, whereas the Kruskal-Wallis test was carried out to compare more than two groups. The X-tile software was applied to categorize patients into two subgroups to reduce the computational batch effect (21). Survival curves were plotted *via* the Kaplan-Meier log rank test. The chi-square test was performed to correlate the ICI score subgroups with somatic mutation frequency, and the Spearman analysis computed the correlation coefficient. CIBERSORT algorithm results with p < 0.05 were adopted for further analysis. Two-tailed p < 0.05 deemed statistical significance. R software (version 4.0.3) was utilized for all statistical analyses.

## Results

### Landscape of the Tumor Immune Microenvironment in Breast Cancer

**Figure 1:**
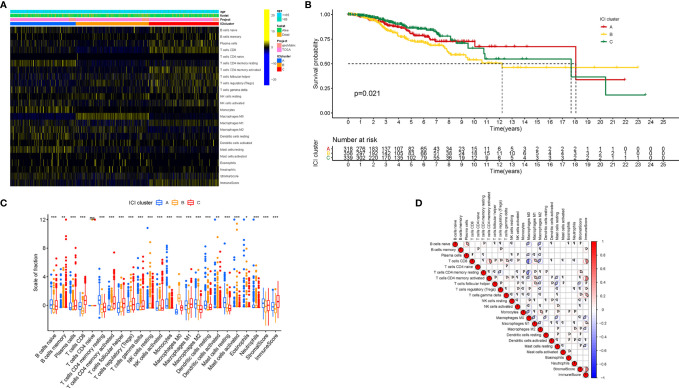
The Landscape of Immune Cell Infiltration in the TIME context. **(A)** Unsupervised clustering of tumor-infiltrating immune cells in BRCA patients. Rows represent tumor-infiltrating immune cells, and columns represent samples. **(B)** Kaplan-Meier curves for the overall survival (OS) of all BRCA patients in discrete ICI clusters. **(C)** The subpopulation of infiltrating immune cells, immune score, and stromal score in three ICI clusters. **(D)** Intrinsic connection of the infiltrating immune cells and immune scores. The asterisks represented the statistical p value (*P < 0.05; **P < 0.01; ***P < 0.001), ns, No Significance.

To further evaluate the subpopulations of inflammatory cells and enrichment of immune signature in BRCA (Tables S1 and 2), the ESTIMATE and CIBERSORT algorithms were performed. Based on the ICI patterns of 1,029 BRCA samples from TCGA-BRCA and GSE58812, the R package “ConsesusClusterPlus” was employed to cluster the BRCA samples into discrete subgroups. According to similarities exhibited in the ICI profiles, we found that k = 3 had an optimal clustering stability. An increasing trend of the cumulative distribution function (CDF) value was considered as an indicator of an outstanding clustering (**Figures S1A–F**). Thus, three different ICI patterns were finally identified by using an unsupervised clustering, including pattern A (318 samples), pattern B (356 samples), and pattern C (339 samples). These patterns were termed as ICI cluster A, ICI cluster B, and ICI cluster C, respectively. The involvement of the ICI patterns with the clinical phenotypes was explored and depicted in the comprehensive heatmap (**Figure 1A**). Kaplan-Meier survival analysis of three distinct ICI patterns indicated that ICI cluster A exhibited a prominent advantage of median survival time, whereas ICI cluster B presented with the worst prognosis (**Figure 1B**, P = 0.021). To further reveal the potential connection between immune scores and infiltrating immune cells, the correlation was presented to visualize the comprehensive landscape of TIME (**Figure 1C**).

### Immune Cell Infiltration Patterns Characterized With Different Immune Phenotypes

Next, characterizing the TIME landscape with CIBERSORT and ESTIMATE was performed to compare the relative subpopulations of infiltrating immune cells and immune scores among three ICI patterns (**Figure 1D**). Cluster ICI-A was associated with a high level of such quiescent cell subpopulations as resting memory CD4 T cells, naive B cells, resting mast cells, resting dendritic cells, and higher stromal score. Existing researches highlighted that the immune-excluded phenotype was characterized by an abundant infiltration of immune cells, which could not penetrate the tumor parenchyma because of the retention of the stroma surrounding cancer nests (22). For that, we surmised that overexpressed stromal elements will weaken the antitumor immunotherapeutic efficacy in the ICI cluster A with a high stromal score. Patients from ICI-B cluster exhibited a remarkably lower infiltration of most immune cells and lower immune score. Thus, ICI cluster B could be considered as decreased-immunogenicity tumors with a weakened immune cell infiltration. The samples in the ICI-C cluster were marked by a significant increase in the subpopulation of the Antitumor lymphocyte cell subsets, such as plasma cells, memory B cells, CD8 T cells, follicular helper T cells, activated memory CD4 T cells, and M1 macrophages. Consistently, ICI cluster C was found to be characterized by remarkably highest immune scores. Abundance of tumor-infiltrating lymphocyte (i.e., activated CD4+/CD8+ T cells, etc.) was significantly associated with the condition of antitumor immunity (23, 24), suggesting that ICI cluster C could be regarded as inflamed tumors. As a result, ICI cluster A was characterized by innate immune cells infiltration but stromal activation and considered as an immune-excluded phenotype; ICI cluster B was characterized by a weakened immune cell infiltration and identified as an immune-desert phenotype; and ICI cluster C was characterized by an elevated inflammation response and recognized as an immune-inflamed phenotype.

**Figure 2:**
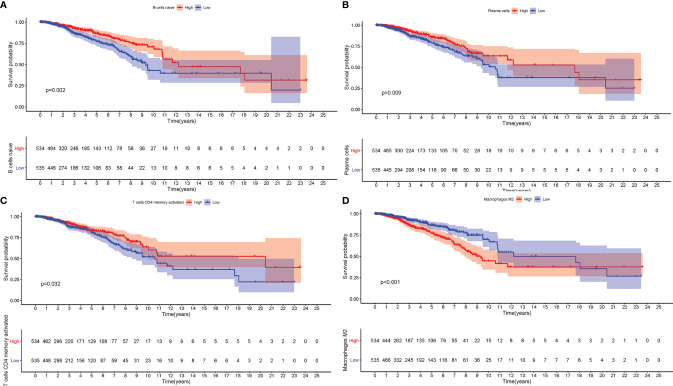
The Role of Immune Cell Infiltration in Prognosis. The overall survival analysis between patients with different infiltration levels of Naïve B cells **(A)**, Plasma cells **(B)**, activated memory CD4 T cells **(C)**, and M2 Macrophages **(D)**.

To further estimate the prognostic value of ICIs, stratification survival analysis was analyzed based on distinct ICIs subpopulations. Notably, infiltration of naive B cells, activated CD4 memory cells, and plasma cells were discovered to be significantly correlated with a longer survival time (**Figures 2A–C**, P < 0.05). By contrary, high density of M2 Macrophages suggested a poor prognosis (**Figure 2D**, P < 0.001). Interestingly, the results showed that immunosuppressive cells subtypes (gamma delta T cells, T regulatory cells) were also enriched in ICI cluster C, which required further interpretation in subsequent analysis.

**Figure 3:**
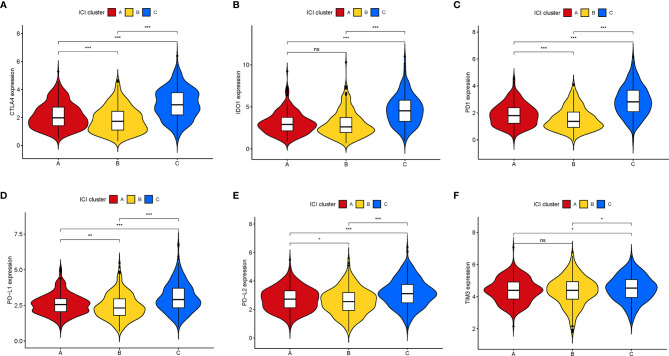
Comparison of ICB-relevant key genes among distinct ICI clusters. The expression levels of CTLA4 **(A)**, IDO1 **(B)**, PD1 **(C)**, PD-L1 **(D)**, PD-L2 **(E)**, and TIM3 **(F)** of patients from distinct ICI clusters. The asterisks represented the statistical p value (*P < 0.05; **P < 0.01; ***P < 0.001), ns, No Significance.

Furthermore, we compared the expression levels of six key immune checkpoints blockade (ICB)-related genes (CTLA4, IOD1, PD1, TIM3, PD-L,1 and PD-L2) among the three ICI clusters. We observed that ICI-C cluster was marked by a significantly highest expression value of ICB-related genes, whereas ICI-B cluster presented with the lowest expression level (**Figures 3A–F**). These results suggested that ICI-C cluster might be more suitable for immunotherapy.

### Identification of Immune Gene Subgroups

**Figure 4:**
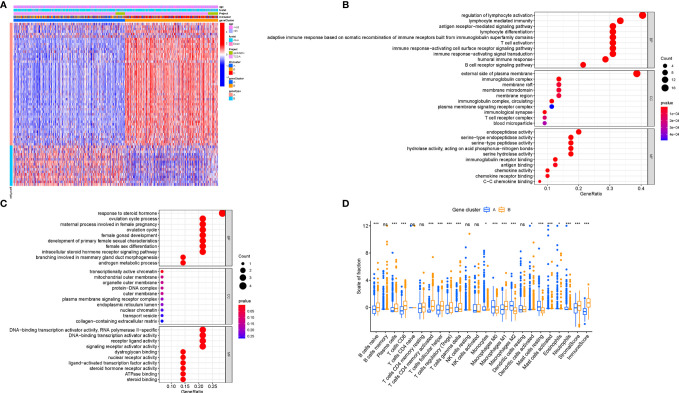
Generation of Immunogenic Gene Subtypes. **(A)** Unsupervised clustering of common DEGs among three ICI cluster groups to categorize patients into two subgroups: gene clusters A–B. **(B, C)** Gene Ontology (GO) enrichment analysis of the two ICI-relevant signature genes: ICI signature genes A **(B)** and B **(C)**. **(D)** The proportion of infiltrating immune cells, stromal score, and immune score in two gene clusters. The asterisks represented the statistical p value (*P < 0.05; ***P < 0.001), ns, No Significance.

To explore the potential ICI-related transcriptional expression alterations across different ICI patterns, we determined 65 DEGs in the combination cohort (TCGA-BRCA and GSE5812) using the limma package, which were considered as the crucial distinguishing index of different ICI phenotypes (Table S3). To better investigate the underlying molecular mechanism, unsupervised clustering analyses were performed based on the obtained 65 most representative ICI phenotype-related genes to stratify the samples into distinct transcriptomic phenotypes (gene clusters A–B; **Figures S2A–F**). ICI-A gene signature including 49 DEGs held a positive correlation with the gene cluster, whereas other 16 DEGs were introduced into the ICI-B gene signature (Table S4). A heatmap was delineated to display the genomic distinction among these different gene subtypes (**Figure 4A**). The significant enrichment of biology was explored and delineated by the GSEA analysis (**Figures 4B, C**), of whose comprehensive details were recorded in Table S5. To investigate the prognostic significance of the ICI gene clustering, the survival analysis was performed by plotting the Kaplan-Meier curve. There was no significant distinction between two gene clusters (p = 0.207; **Figure S3A**), however, the median survival time of gene cluster B was longer than gene cluster A.

To elucidate the potential role of distinct gene clusters in TIME contexture, the relative subpopulations of infiltrating immune cells were estimated with the ESTIMATE algorithm and CIBERSORT approach. The gene cluster A was closely correlated with resting mast cells, M0 and M2 Macrophages, monocytes, neutrophils, and eosinophils, corresponding to an immunosuppressive phenotype (25, 26). Conversely, the gene cluster B showed an increased infiltration of CD8 T cells, plasma cells, activated memory CD4+ T cells, gamma delta T cells, follicular helper T cells, and M1 Macrophages, which was termed as the active-immune phenotype (27, 28). Interestingly, gene cluster B obtained a higher immune score, which insinuates an immunologically “hot” phenotype (**Figure 4D**).

**Figure 5:**
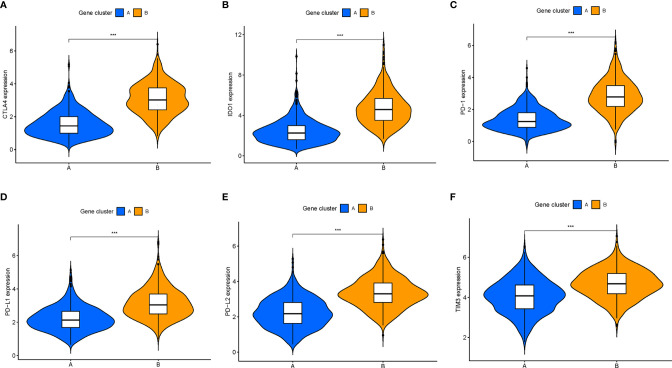
Comparison of ICB-relevant key genes among distinct ICI gene clusters. The expression levels of CTLA4 **(A)**, IDO1 **(B)**, PD1 **(C)**, PD-L1 **(D)**, PD-L2 **(E)**, and TIM3 **(F)** of patients from distinct ICI gene clusters. The asterisks represented the statistical p value (***P < 0.001).

Besides, there was a significant difference of the expression levels of ICB-related genes between two gene clusters (all p < 0.001; **Figures 5A–F**). The ICI-B gene cluster was significantly marked with higher expression levels of ICB relevant genes compared with the ICI-A gene cluster, indicating that the ICI-B gene cluster may obtain a benefit from immunotherapy.

### Validation of the Immune Cell Infiltration Score in Breast Cancer

**Figure 6:**
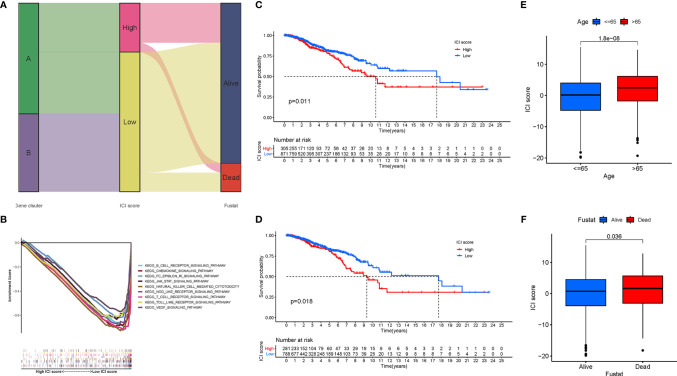
Development of the ICI Scores. **(A)** Sankey diagram of ICI gene cluster distribution in subgroups with distinct ICI clusters, ICI scores, and survival status. **(B)** Enrichment plots showing B cell receptor, chemokines, JAK/STAT, Nod like receptor, and VEGF signaling pathways in the low ICI score subgroup. Kaplan-Meier curves for the high and low ICI score groups in the TCGA-BRCA cohort **(C)** and GSE58812 cohort **(D)**. **(E)** Comparison of ICI score between young (<=65) and old (>65) subgroups. **(F)** Comparison of ICI score between dead and alive subgroups.

Although the potential roles of the ICI patterns in prognostic prediction and TIME information were revealed, these above analyses only placed an emphasis on the sample population, which was not able to be accurately performed in individuals. Given the high complexity and heterogeneity of ICI, a scoring system, termed as the ICI score, were developed based on these ICI phenotype-related signature genes to investigate the ICI patterns of individual patients. Sankey diagram represented the distribution of the patients in two different gene clusters (**Figure 6A**). To further explore the potential role of the ICI score in the biological processes, GSEA was carried out in the low‐ as well as the high‐ICI score subgroup, from which we could discover that the JAK/STAT and VEGF signal pathways were evidently activated in patients with a low ICI score (**Figure 6B**). Additionally, the prognostic value of the ICI score in predicting the overall survival time was determined by classifying the patients into high- or low-ICI score groups. As expected, low-ICI score suggested a prominent prognosis advantage in both the combination cohort (P = 0.011, **Figure 6C**) and TCGA dataset (P = 0.018, **Figure 6D**). In the GSE58812 cohort, patients with a low ICI score had a higher survival probability relative to patients with a low ICI score (P = 0.293; **Figure S3B**). In addition, with an accumulated age (P = 1.8e-08, **Figure 6E**), the ICI score was significantly elevated. Moreover, dead survival status was significantly associated with a higher ICI score (P = 0.036, **Figure 6F**). Stratification analysis was performed to validate whether the ICI score still had a powerful prognostic predictive ability when BRCA patients were classified into various subgroups based on the clinical characteristics. Relative to patients with a low-ICI score, high-ICI score patients presented a poorer prognosis in young (age< = 65) subgroups (**Figures S3C–F**).

### The Correlation of the Prognostic Immune Cell Infiltration Score With the TIME Context in Breast Cancer

**Figure 7:**
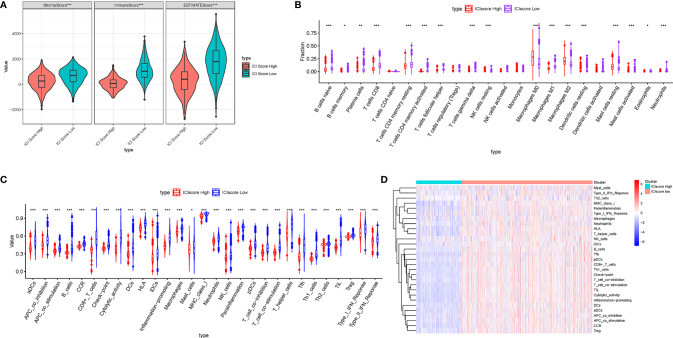
Correlation of ICI score with the characterization of TIME. **(A)** Comparison of the ESTIMATE algorithm (estimate score, stromal score, and immune score) between patients who obtained different ICI scores. **(B)** Difference of infiltrating immune cell subpopulations and levels between the low-/high-ICI score groups. Violinplot **(C)** and heatmap **(D)** of distinction of enrichment of immune-related signatures between the ICI score-low and ICI score-high groups. The asterisks represented the statistical p value (*P < 0.05; **P < 0.01; ***P < 0.001).

Considering that the ICI patterns were derived from the infiltration of immune cells, we further explored the potential contribution made by the ICI score in the complexity and diversity of TIME contexture. Firstly, we observed that the low-ICI score patients obtained a higher estimate score, immune score, and stromal score than the high-ICI score patients (**Figure 7A**). The result showed that the ICI score was negatively and remarkably associated with antitumor lymphocyte cell subpopulations such as matured memory CD4 cells, CD8 T cells, M1 Macrophages, and neutrophils, whereas an experienced positive correlation with the abundance of resting mast cells, M0 and M2 Macrophages (**Figure 7B**). The ssGSEA results presented that all of the fraction of immune cells infiltration and enrichment of immune signatures were significantly higher in patients with a low ICI-score (**Figure 7C**). Heatmap shows each patient with a corresponding enrichment of the immune signature from the low-/high-ICI score subgroups (**Figure 7D**). Based on these results, it was discovered that distinct ICI score samples exhibited a significantly different ICI characterization. Low-ICI score subjects were characterized by an immune-excluded condition (stromal activation and abundant immune infiltration), while samples with a high-ICI score were corresponding to an immune-desert contexture, characterized by a decreased immune infiltration. These findings highlighted that the ICI score might serve as nonnegligible players in an immunological cross-talk of TIME.

### The Correlation of the Immune Cell Infiltration Scores With Immunotherapy

**Figure 8:**
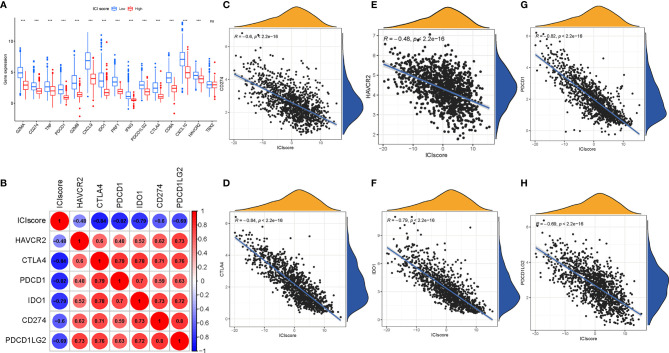
Immunotherapeutic Significance of ICI Scores. **(A)** ICB-relevant genes (CD274, CTLA4, HAVCR2, IDO1, PDCD1, and PDCD1LG2) and inflammatory-related genes (CD8A, CXCL10, CXCL9, GZMA, GZMB, IFNG, PRF1, TBX2, and TNF) expressed in the high and low ICI score subgroups. Correlation between the ICI score with crucial immune checkpoint blockade genes. **(B)** Correlation analysis between immune checkpoint inhibitors (CD274, PDCD1, PDCD1LG2, CTLA4, HAVCR2, and IDO1) with the ICI score. **(C)** Correlation between the ICI score and CD274. **(D)** Correlation between the ICI score and CTLA4. **(E)** Correlation between the ICI score and HAVCR2. **(F)** Correlation between the ICI score and IDO1. **(G)** Correlation between the ICI score and PDCD1. **(H)** Correlation between the ICI score and PDCD1LG2. The asterisks represented the statistical p value (***P < 0.001), ns, No Significance.

To estimate the tolerance condition and immune activity of the low-/high-ICI Score subgroups, ICB-relevant genes (CD274, CTLA4, HAVCR2, IDO1, PDCD1, and PDCD1LG2) and inflammatory-related genes (CD8A, CXCL10, CXCL9, GZMA, GZMB, IFNG, PRF1, TBX2, and TNF) were introduced into an expression level analysis (29, 30). As depicted in **Figure 8A**, 14 out of 15 expression levels of immune-activity and tolerance-condition related genes were significantly upregulated in patients with a low-ICI score (P < 0.001).

Moreover, we correlated six ICB key targets (PDCD1, CD274, PDCD1LG2, CTLA‐4, HAVCR2, and IDO1) and ICI score to reveal its potential player in the ICB treatment of BRCA (**Figure 8B**). We found that the ICI score was significantly negatively correlated with CD274 (R = -0.6, p < 0.001), CTLA4 (R = -0.84, p < 0.001), HAVCR2 (R = -0.48, p < 2.2e−16), IDO1 (R = -0.79, p < 0.001), PDCD1 (R = -0.82, p < 0.001), and PDCD1LG2 (R = -0.69, p < 0.001; **Figures 8C–H**), implying that the ICI score might act as a nonnegligible role in the prediction of responsiveness to ICB treatment in patients with BRCA.

**Figure 9:**
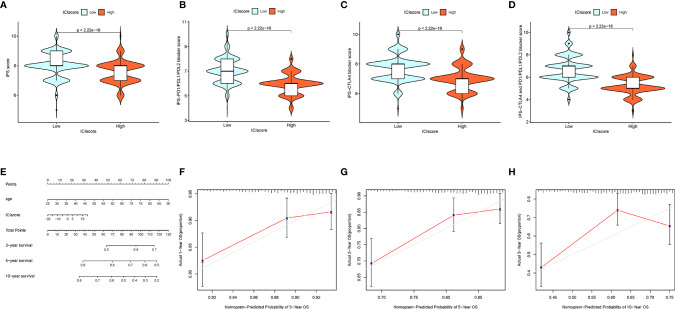
The Estimation of the ICI Score in Immunotherapy Response. **(A)** IPS score distribution plot. **(B)** IPS–CTLA4 blocker score distribution plot. **(C)** IPS–PD1/PDL1/PDL2 blocker score distribution plot. **(D)** IPS–CTLA4 and PD1/PDL1/PDL2 blocker score distribution plot. **(E)** Nomogram was assembled by age and risk signature for predicting survival of BRCA patients. **(F)** Three‐year nomogram calibration curves of the combination of the TCGA and GEO cohorts. **(G)** Five‐year nomogram calibration curves of the combination of the TCGA and GEO cohorts. **(H)** Ten‐year nomogram calibration curves of the combination of the TCGA and GEO cohorts.

To predict immunotherapeutic outcome under ICI scores, two subtypes of IPS values (IPS-PD-1/PD-L1/PD-L2 positive and IPS-CTLA-4 positive) were implemented as the surrogates of the responses of BRCA patients to immunotherapy. In our predictive scheme, IPS score, IPS–CTLA4 blocker score, IPS–PD1/PDL1/PDL2 blocker score, and IPS–CTLA4 and PD1/PDL1/PDL2 blocker score were higher in low-ICI score samples (all P < 2.22e−16; **Figures 9A–D**), highlighting that BRCA samples with a low ICI score might be suitable for immunotherapy.

### Drawing of the Prognostic Nomogram

Based on the stepwise Cox regression model, BRCA samples with clinical data from both the TCGA dataset and GSE58812 were employed to construct a prognostic nomogram predicting the 3-, 5-, and 10-year OS. To estimate the OS rate of the individual patients quantitatively, a prognostic nomogram consisting of the ICI score and age was constructed (**Figure 9E**). Calibrate curves was plotted to demonstrate a great prognostic predictive validity of the OS in the as-constructed nomogram plot (**Figures 9F–H**).

### The Association Between the Immune Cell Infiltration Scores With the Tumor Mutation Burden

**Figure 10:**
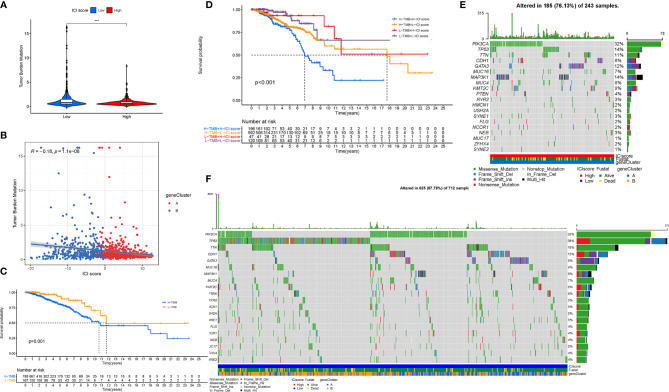
The Correlation between the ICI Score and TMB. **(A)** Difference of TMB between patients from the low-/high-ICI score subgroups. **(B)** Scatterplots depicting the negative correlation between the ICI scores and TMB. **(C)** Kaplan-Meier curves for the high and low TMB groups. **(D)** Kaplan-Meier curves for patients stratified by both TMB and ICI scores. The oncoPrint was constructed using the high ICI scores **(E)** and low ICI scores **(F)**. The asterisks represented the statistical p value (***P < 0.001).

Existing studies have contributed a strong evidence to demonstrate that a high tumor burden mutation (TMB) was correlated with the increasement of infiltrating CD8+ T cells, which recognized tumor neoantigens then resulted in intense tumor-killing effects to annihilate tumor cells (31–33).Thus, we speculated that TMB might act as a prognostic factor of responsiveness to antitumor immunotherapy and aimed to investigate the potential interaction between the ICI scores and TMB to uncover the hereditary variations of the ICI score subtype. Firstly, the TMB level was detected both in the low and high ICI score subgroups. We observed that the higher TMB concentrated on the subgroup with an ICI-low score (p < 0.001, **Figure 10A**). Subsequent correlation analysis further validated that the TMB was negatively and significantly correlated with the ICI score (R = -0.18, p = 1.1e−08; **Figure 10B**). Then, the patients were assigned into distinct subtypes on the line of the TMB immune set point, as stated before (34). Taking advantage of the Kaplan-Meier analysis, we demonstrated that a low TMB suggested a higher survival probability (**Figure 10C**, p = 0.001). To further explore the validity of the consistent prognostic significance of the ICI scores and TMB, we validated the cooperative effect of two indicators in the prognostic prediction of BRCA. As demonstrated in a stratified survival curve, there was no interference of the TMB status with the ICI scores in the prognostic predictive performance. ICI score subgroups exhibited evident prognosis distinctions in both low and high TMB status subtypes (p < 0.001; **Figure 10D**). In summary, these results suggested that the ICI score might act as an independent prognostic predictor and hold the potential to evaluate the clinical outcome of an antitumor immunological treatment.

Besides, we analyzed the distribution of gene mutation in BRCA between the low and high ICI score subtypes. The comprehensive landscape of somatic variants visualized the mutation patterns and clinical features of the top 20 driver genes with the most frequent alteration (**Figures 10E, F**). The significantly mutated gene (SMG) mutational landscapes presented that MAP3K1 (14% *vs.* 6%) experienced higher somatic mutation rates in the high ICI core subtype, while TP53 (38% *vs.* 14%) possessed higher somatic mutation rates in the low ICI score subgroup. These findings might contribute a novel insight into the intrinsic connection of ICI abundance and somatic variants in the immunotherapy of BRCA.

## Discussion

Breast cancer is regarded as a devastating malignant tumor and the primary reason for cancer-related death of women in most countries. The high heterogeneity from the phenotypes of tumor microenvironment and the genotypes of cancer cells was the main cause for diversity of different sensitivity to clinical treatments and distinct clinical outcomes. The TNM staging was used widely to guide for clinical management and predict prognosis in clinical application. However, it could not precisely predict the clinical outcome of patients with the similar clinical variables (35). Thus, it is required to urgently identify the promising and effective indicators for the survival evaluation and treatment option in BRCA.

Immune-based antitumor treatment has made a great breakthrough in multiple human cancers and reignited the enthusiasm of people of immunotherapeutic strategy for the clinical intervention in BRCA (36, 37). Currently, encouraging clinical success have demonstrated an improved immunotherapeutic efficacy in a minority of advanced BRCA patients (38). Mounting studies have highlighted that the regulation of the infiltrating immune cells could serve as the determining drivers in tumor progression and anti-tumor immunity (39–41). As plenty of researches concentrated on single TIME subset or several immune-related genes (42–45), the comprehensive landscape of TIME mediated by an integrated modification mediated by distinct ICI subpopulations have not been comprehensively illustrated in BRCA.

Herein, our findings demonstrated that escalated infiltrations of plasma cells, activated CD4 memory cells, and naive B cells, and downregulation of M2 Macrophages abundance were remarkably correlated with a better overall survival. This suggested that the immune activity condition served as an opposing role in tumor progression and positively affects the clinical outcome of immune-based strategies. Accumulating data has pointed out that TIME of breast tumor presented high levels of infiltrating immune cells (46). However, Ib phase clinical trial of Avelumab reported that the overall response rate (ORR) for the entire BRCA cohort was only 4.8% (47). This implies that the immune phenotypes cannot precisely assess the outcome of immunotherapy in BRCA due to its heterogeneity.

In addition, we analyzed the ICI profiles of 1,198 BRCA patients from the TCGA-BRCA cohort and GSE58812 dataset, then classified these samples into three distinct ICI subgroups by employing a consensus clustering. Three different ICI patterns associated with distinct immune phenotypes were identified, which were characterized by diverse anti-cancer immunology. The ICI cluster-A experienced an abundant infiltration of quiescent immune cells with abundant stromal elements, which could be considered as an immune-excluded phenotype (22). Although a high infiltration of immune cells was presented in the immune-excluded phenotype, these immune cells, the penetration of which into the parenchyma of the tumor was impeded by the abundant stromal element, were unable to function as recognition and elimination of cancer cells. In contrast, the ICI cluster B, corresponding to an immune-desert phenotype, was characterized with the absence of infiltrating immune cells and weaken immune activity. Finally, the ICI cluster C was marked with the inflammatory condition of TIME, which was regarded as an immune-inflamed phenotype. The immune-inflamed phenotype, also known as a hot tumor, is characterized with immune activation and abundant immune cell infiltration. However, patients with this ICI pattern did not present a matching survival advantage. Considering a significant upregulation of ICB key genes in the ICI cluster C subtype, this ICI pattern may be affected more by immune checkpoint blockade pathways. Therefore, we speculated that immune evasion mediated by ICB in cluster C inhibited the antitumor effect of immune cells. Based on the TIME characteristics in each subtype, it supported the robustness of the clustering of immune phenotypes with distinct ICI patterns.

Therefore, we combined the ICI patterns and immune-related genes expression profiles to reveal the comprehensive properties of TIME, which might be an effective and robust tool in an individual tailored treatment with a further advanced precision immunotherapy. The molecular feature which regulated the immune activity of BRCA-TIME was our primary concerns, we first extracted the immune-correlated genes on the basis of the ICI gene clusters. Between two different clusters, the ICI gene-B subgroup obtained a higher immune score, and several densities of anti-tumor cells (i.e., plasma cells, M1 Macrophages, CD8+ T cells, activated CD4+ T cells, etc.), which was termed as activated immune phenotype (48, 49). However, patients with this from this gene cluster did not show a matching survival advantage. To our surprise, a higher stromal score was enriched in the ICI gene-B cluster, indicating that stromal activation inhibited the antitumor effect of immune cells. By contrary, the ICI gene-A cluster had relatively low immune scores and immune-response-related cell abundance, suggesting an immune-exhausted condition in this cluster. Taken together, these findings uncovered that the stromal activation in the ICI gene-B cluster might suppress an effective antitumor immune response of abundant and activated immune cell infiltration, while the “immune-exhausted phenotype” in the ICI gene-A cluster might lead to immune evasion and immunotherapy resistance. Moreover, the ICI gene-B cluster experienced upregulated ICB-related genes, suggesting samples from the ICI gene-B cluster were more likely to respond to immunotherapy.

Due to the patient-specific heterogeneity of the TIME context, there was an urgent demand to analyze the ICI patterns quantitatively in each patient. Herein, we determined a novel individual-based signature derived from subtype-specific markers and calculated a score to reveal the landscape of ICI quantitatively. As such, ICI-based scoring scheme (ICI score) were established to quantify the distinct ICI patterns, directing a precision immunotherapeutic intervention. The powerful prognostic value of the ICI score was validated by the K-M survival analysis. Moreover, a great clinical significance of the ICI score was verified by analyzing the distribution differences. Finally, a novel and robust prognostic age-ICI score nomogram for clinical application was established to predict the individual sample prognosis quantitatively.

To further the role of the ICI score in the diversity and complexity of TIME, we compared the ICI patterns between the low and high ICI score subgroups by employing the CIBERSORT, ESTIMATE, and ssGSEA algorithms. We observed that almost all immune cells infiltration, immune scores, and immune-relevant signatures were significantly and negatively correlated with the ICI scores, implying that the ICI score might serve as an immunosuppressive indicator. The immune-desert ICI pattern obtained a higher ICI score, while the pattern corresponding to the immune-excluded phenotype exhibited a lower ICI score. Furthermore, we validated that the ICI score were significantly and negatively correlated with the ICB treatment vital targets (i.e., PDCD1, etc.), suggesting that a low ICI score might be more sensitive to immunotherapy. In consistent, the upregulated values of immunophenoscore, such as IPS–PD1/PDL1/PDL2 blocker score and IPS–CTLA4 blocker score, indirectly suggested the higher tumor immunogenicity for subjects in the low-ICI score samples. These findings suggested that the landscape of the ICI patterns may contribute a novel insight into the ICB therapy efficacy prediction in patients with BRCA. In the absence of the ICB treatment dataset in the BRCA cohort, we were unable to investigate the relationship between the ICI score and ICB immunotherapy response. Notwithstanding, further clinical validation is demanded for these results at a larger cohort and different centers.

Taken together, a low-ICI score experienced the abundance of immune infiltration, which were impeded against by the presence of stromal components and higher stromal score. It was well-established that the activation of the TGF-β- and EMT-related pathways inhibited the permeation of immune cells (50). Specific inhibitors interrupting the TGF-β related pathways have been discovered to restore the anticancer immunity and reprogram context of TIME (51, 52). In addition, GSEA analysis exhibited that a low-ICI score showed a significant enrichment in the pathways, like the VEGF signal transduction pathway and so on. Recent researches highlighted that the vascular endothelial growth factor (VEGF) signaling pathway, a crucial regulator of angiogenesis, is critical for the progression and distant dissemination of cancer and could be employed as a potential antiangiogenic target in BRCA (53). Based on the above findings, BRCA patients with a low-ICI score were speculated to benefit from combination administration with TGF-β blockade and VEGF-targeting agents.

Currently, several clinical data pointed out a correlation between the genetic alternations with responsiveness to the immunological treatment (54, 55). We calculated and determined the TMB, which is a predictive indicator of sensitivity to immunological treatment, decreased significantly with elevated ICI scores. In this work, the TP53 mutation rates were revealed to be markedly augmented in the low-ICI score subtype, while the mutation rate of the SMGs of MAP3K1 was increased in the patients with a high-ICI score. Based on published articles, mutation of MAP3K1 was potential drug targets in combination with MEK inhibitors in breast tumors (56). Recent studies indicated that TP53, of which mutation was identified a potential biomarker role in BRCA, is one of the most frequently mutated genes (57). The distribution differences of the ICI score-related mutated driver genes were significantly correlated with the anti-tumor immunity, highlighting the complicated interaction of the ICI patterns with somatic mutation contributed into tumor immunogenomic regulation. Subsequent a stratified survival curve demonstrated that the ICI scores held a prognostic predictive capability which was independent of TMB, suggesting that TMB and ICI score represent different aspects of immunobiology. Besides, the ICI score together with the mutation data revealed the significant distinction of genes variant frequency between the high and low ICI score groups from the level of genome.

In this work, diverse ICI patterns among 1,198 BRCA samples based on 22 ICI subpopulations were comprehensively identified. Additionally, the complexity and heterogeneity of individual tumor immune microenvironment, a critical foundation for the regulation of anti-tumor immunity, were comprehensively dissected with distinct ICI patterns. Moreover, the ICI scoring scheme was constructed to quantify the ICI pattern of individual sample, and a prognostic age-ICI score nomogram was established to estimate the prognosis of BRCA patients quantitively. Furthermore, we have demonstrated the complicated correlation and synergistic effect between the ICI score and the somatic mutation. In conclusion, the comprehensive evaluation of the ICI patterns in individual tumor will contribute a novel insight into delineating landscape of TIME and directing precision immunotherapeutic strategy.

## Data Availability Statement

The original contributions presented in the study are included in the article/**Supplementary Material**. Further inquiries can be directed to the corresponding author.

## Author Contributions

WH designed the overall study and revised the paper. QX drafted the manuscript and performed the data analysis. SC and YH participated in the data collection. All authors contributed to the article and approved the submitted version.

## Funding

This study was supported by Funding of Wenzhou Municipal Science and Technology Bureau (Grant No. Y2020971).

## Conflict of Interest

The authors declare that the research was conducted in the absence of any commercial or financial relationships that could be construed as a potential conflict of interest.

## Publisher’s Note

All claims expressed in this article are solely those of the authors and do not necessarily represent those of their affiliated organizations, or those of the publisher, the editors and the reviewers. Any product that may be evaluated in this article, or claim that may be made by its manufacturer, is not guaranteed or endorsed by the publisher.

## Abbreviations

BRCA, breast cancer; CD274,Also known as PD-L1; DCs, dendritic cells; DEG, differentially expressed genes; DEL, deletion; FDR, false discovery rate; GEO, Gene Expression Omnibus; GO, Gene ontology; GSEA, Gene set enrichment analysis; ICB, immune checkpoint blockade; ICI, immune cell infiltration; INS, insertion; KEGG, Kyoto Encyclopedia of Genes and Genomes; K-W, Kruskal-Wallis; MAF, Mutation Annotation Format; OS, overall survival; PDCD1,Also known as PD-1; PDCD1LG2,Also known as PD‐L2; ROC, receiver operating characteristic; SNP, single nucleotide polymorphism; ssGSEA, single-sample gene set enrichment analysis; TCGA, The Cancer Genome Atlas; TICs, tumor‐infiltrating immune cells; TILs, Tumor Infiltrating Lymphocytes; TIME, tumor immune microenvironment; TMB, tumor mutation burden; TNM, Tumor Node Metastasis.
