# Supplementary material for: Landscape of Immune Microenvironment Under Immune Cell Infiltration Pattern in Breast Cancer
Source: Front Immunol. 2021 Aug 27;12:711433. doi: 10.3389/fimmu.2021.711433 (PMC8429934; doi:10.3389/fimmu.2021.711433)
Supplement: Supplementary file 1 [file DataSheet_1.docx]

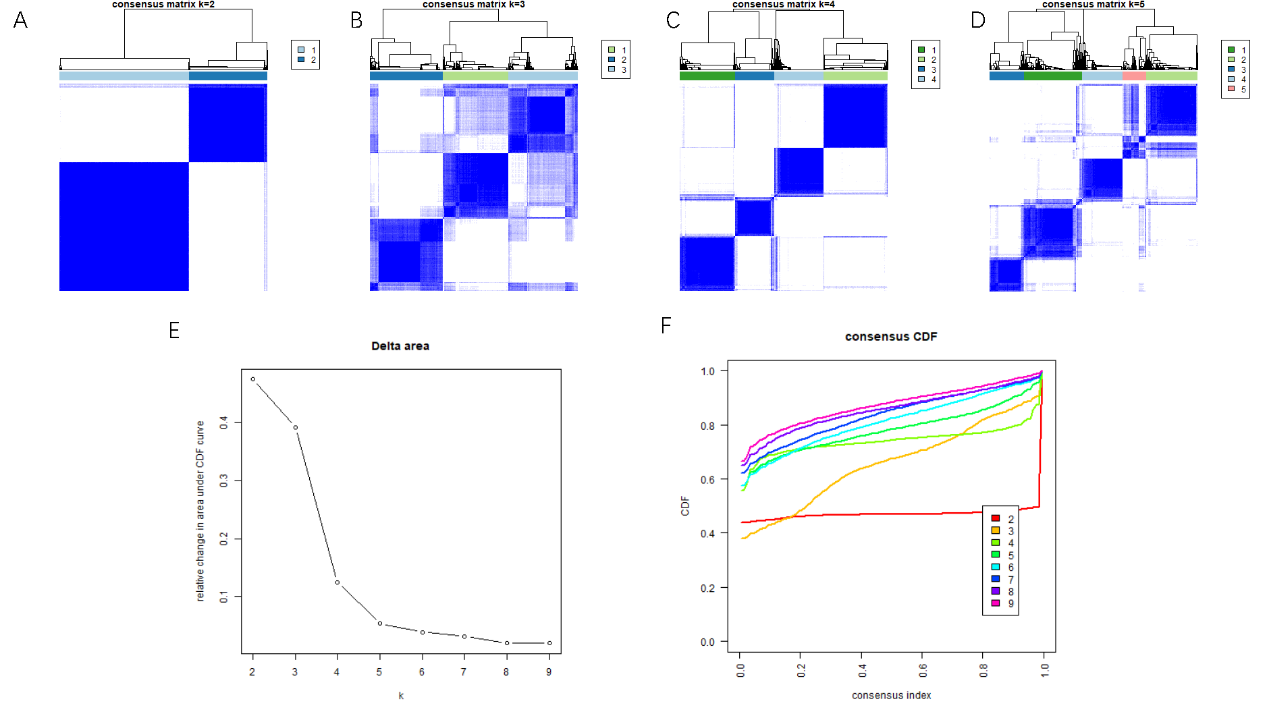


**Figure S1:** Consensus clustering based on the ICI patterns. (A-D) Consensus matrixes of BRCA samples for each k (k = 2–5), displaying the clustering stability using 1000 iterations of hierarchical clustering. (E) The relative change in area under the CDF curve for k = 2–9. (F) Area under cumulative distribution function (CDF) curve when index k ranges from 2 to 10.


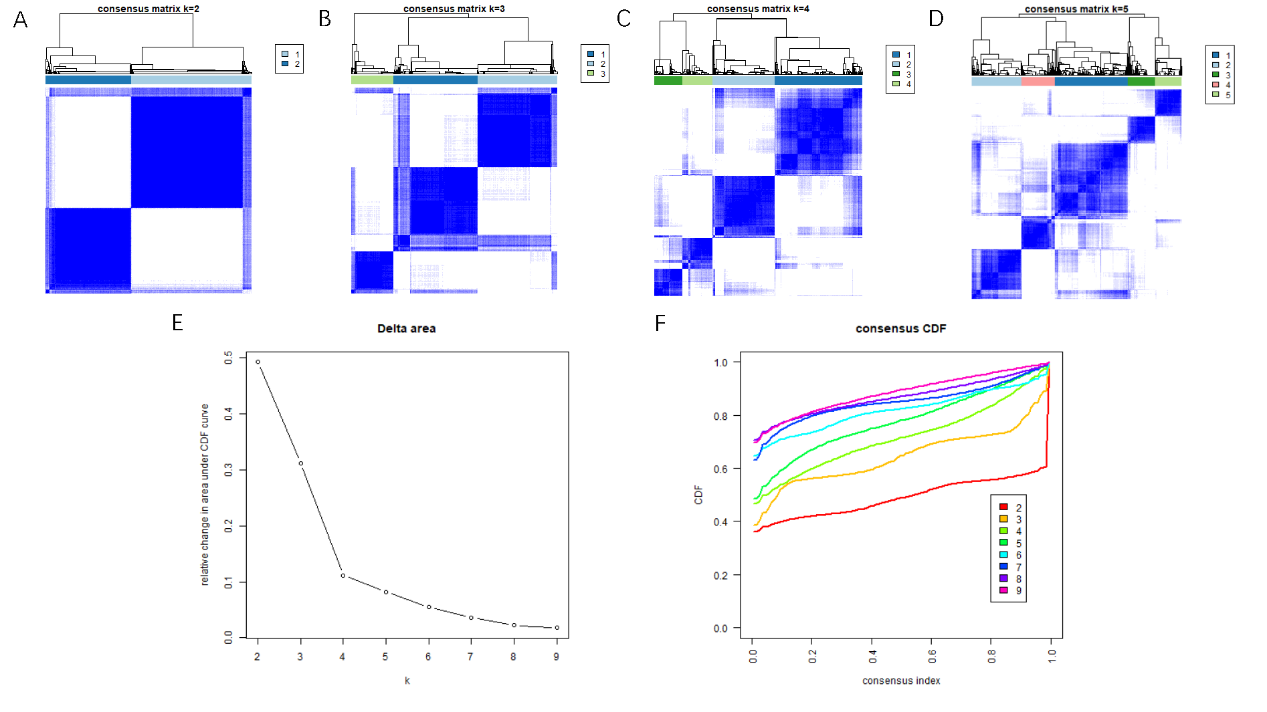


**Figure S2:** Consensus clustering based on DEGs of different ICI clusters. (A-D) Consensus matrixes of BRCA cohorts for each k (k = 2–5), displaying the clustering stability using 1000 iterations of hierarchical clustering. (E) The relative change in area under the CDF curve for k = 2–9. (F) Area under cumulative distribution function (CDF) curve when index k ranges from 2 to 10.


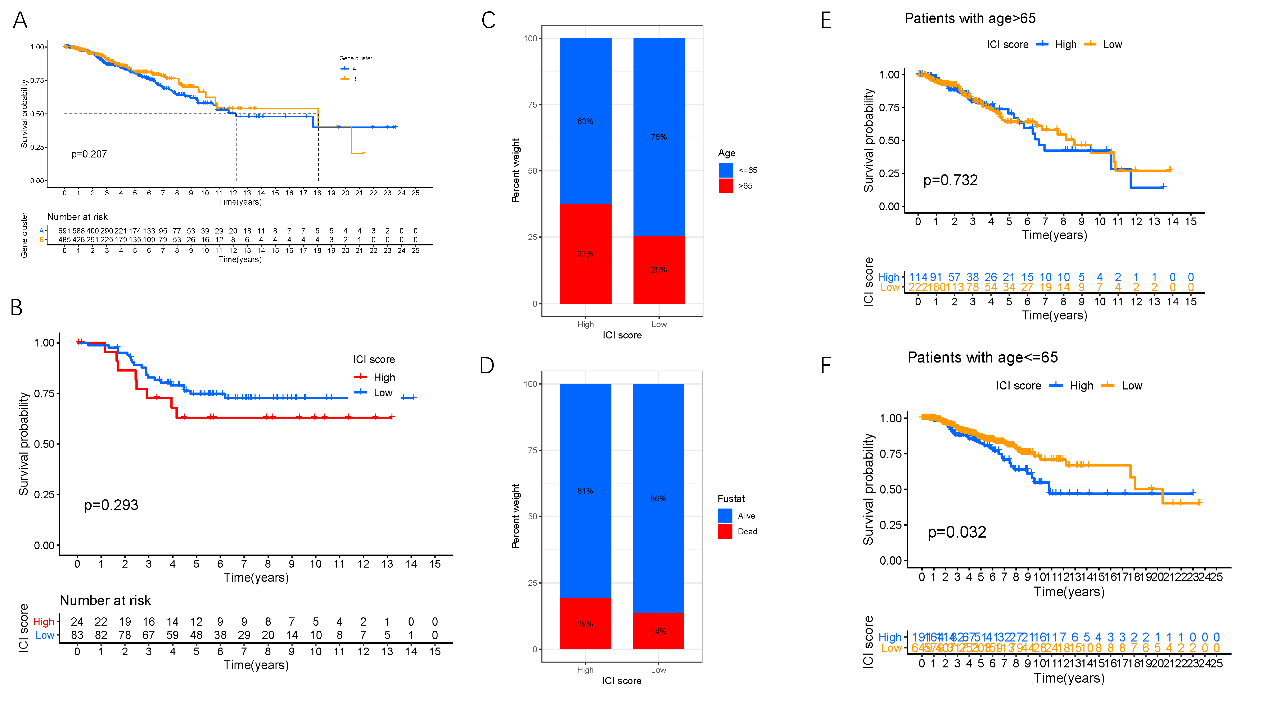


**Figure S3:** (A) Kaplan-Meier plotters for two ICI gene clusters of patients. (B) Kaplan-Meier plotters for different ICI score subgroups in GSE58812. Rate of age (C) and survival status (D) in high or low ICI score groups. (E-F) Kaplan–Meier survival analysis for multiple BRCA subgroups according to ICI score stratified by age.
